# Comparison of behaviorally inhibited and typically developing children’s play behaviors in the preschool classroom

**DOI:** 10.3389/fpsyg.2023.1193915

**Published:** 2023-07-12

**Authors:** Lindsay R. Druskin, Danielle R. Novick, Kelly A. Smith, Andrea Chronis-Tuscano, Nicholas J. Wagner, Stephanie Pham, Hailey M. Fleece, Christina M. Danko, Kenneth H. Rubin

**Affiliations:** ^1^Department of Psychology, West Virginia University, Morgantown, WV, United States; ^2^Department of Psychology, University of Maryland, College Park, MD, United States; ^3^Yale Child Study Center, Yale School of Medicine, New Haven, CT, United States; ^4^Department of Human Department & Quantitative Methodology, University of Maryland, College Park, MD, United States; ^5^Department of Psychological & Brain Sciences, Boston University, Boston, MA, United States; ^6^Department of Counseling, Higher Education, and Special Education, University of Maryland, College Park, MD, United States

**Keywords:** behavioral inhibition, solitude, peer interactions, temperament, anxiety, preschoolers

## Abstract

**Introduction:**

Behavioral inhibition (BI) is a temperamental trait characterized by a bias to respond with patterns of fearful or anxious behavior when faced with unfamiliar situations, objects, or people. It has been suggested that children who are inhibited may experience early peer difficulties. However, researchers have yet to systematically compare BI versus typically developing children’s observed asocial and social behavior in familiar, naturalistic settings.

**Method:**

We compared the in-school behaviors of 130 (*M* = 54 months, 52% female) highly inhibited preschoolers (identified using the parent-reported Behavioral Inhibition Questionnaire) to 145 (*M* = 53 months, 52% female) typically developing preschoolers. Both samples were observed on at least two different days for approximately 60 min. Observers used the *Play Observation Scale* to code children’s behavior in 10-s blocks during free play. Teachers completed two measures of children’s behavior in the classroom.

**Results:**

Regression models with robust standard errors controlling for child sex, age, and weekly hours in school revealed that preschoolers identified as BI engaged in significantly more observed reticent and solitary behavior, and less social play and teacher interaction than the typically developing sample. Children with BI also initiated social interaction with their peers and teachers less often than their counterparts who were not inhibited. Teachers reported that children identified as BI were more asocial and less prosocial than their non-BI counterparts.

**Discussion:**

Significantly, the findings indicated that inhibited children displayed more solitude in the context of familiar peers. Previous observational studies have indicated behavioral differences between BI and *unfamiliar* typical age-mates in *novel* laboratory settings. Children identified as BI did not receive fewer bids for social interaction than their typically developing peers, thereby suggesting that children who are inhibited have difficulty capitalizing on opportunities to engage in social interaction with familiar peers. These findings highlight the need for early intervention for children with BI to promote social engagement, given that the frequent expression of solitude in preschool has predicted such negative outcomes as peer rejection, negative self-regard, and anxiety during the elementary and middle school years.

## 1. Introduction

Behavioral inhibition (BI) is a temperamental style characterized by a bias to respond with vigilant patterns of fearful or anxious responses when exposed to unfamiliar situations, objects, or people (Kagan et al., 1988; Fox et al., 2005). Behavioral inhibition can be reliably measured as early as infancy, and it is estimated that 15–20% of children present with BI (Degnan and Fox, 2007).

Researchers have reported that BI is manifested by a variety of behaviors at different ages. Significantly, researchers have demonstrated that there exists continuity from laboratory assessments of BI in infancy to observed fearfulness in the face of *unfamiliar* objects, adults, and toddlers at two-years, and subsequently to specific forms of social withdrawal expressed in the company of *unfamiliar* peers during the preschool and early elementary school years (e.g., Fox et al., 2001; Rubin et al., 2002; Henderson et al., 2004; Degnan et al., 2008; Kiel and Buss, 2011; Buss et al., 2013; Brooker et al., 2016). Toddlers who have been identified as behaviorally inhibited display fewer smiling, speaking, and approach behaviors than their age-mates who are not inhibited when in the presence of unfamiliar objects and adults (Garcia Coll et al., 1984). During the preschool years, children who are behaviorally inhibited require longer “warm up” periods before approaching or initiating play with unfamiliar children and adults (Kagan et al., 1987). When observed during free play in a laboratory setting with unfamiliar age-mates who are not behaviorally inhibited, inhibited preschoolers have been found to display more *reticent* behavior (watching peers from afar; being unoccupied) and to engage in more solitary activity (Rubin et al., 2002; Henderson et al., 2004) than their non-inhibited age-mates.

As noted above, the extant evidence base has focused largely on BI in the context of novel situations and unfamiliar peers (see Rubin et al., 2009, 2018 for reviews). Researchers have consistently demonstrated links between BI, as assessed during infancy and toddlerhood, and observed displays of social *reticence* in groups of *unfamiliar* peers in preschool-, kindergarten- and early elementary-aged children (e.g., Fox et al., 2001; Rubin et al., 2002; Henderson et al., 2004; Degnan et al., 2008). Furthermore, *in laboratory settings*, elevated BI in early childhood has been shown to predict less observed interpersonal behavior (e.g., the display of fewer positive social initiations/reactions, less time spent in social play) and poorer social skills (e.g., incompetent social problem-solving skills) during the elementary school years (Nelson et al., 2005; Walker et al., 2014; Penela et al., 2015). These findings underscore how the socially avoidant tendencies of inhibited children may impede proficiency in the age-appropriate social skills derived from engagement with peers.

The developmental cascade from BI to social withdrawal has been captured in a conceptual model that has guided much of the current research on the predictors, concomitants, and consequences of BI in infants, preschoolers, and school-age youth (Rubin and Chronis-Tuscano, 2021; see Rubin et al., 1991 for a review). Briefly, within this conceptual model, BI, as assessed in the infant and toddler years, serves as an early predictor of anxiogenic parental behaviors (e.g., oversolicitousness and overprotectiveness). For example, in the case of oversolicitous parenting, parents may interrupt social situations where children would have the opportunity to experience challenges because they may believe that children are unable to navigate social difficulties without parental support (Rubin et al., 1997). Kiel et al. (2015) also identified curvilinear associations between parental encouragement and children’s separation anxiety, such that overly protective maternal behavior or overly encouraging behavior (i.e., to the point of intrusiveness) was related to greater separation anxiety in inhibited children. Moreover, in the aforementioned model it is proposed that the resulting lack of sufficient opportunities to engage in novel social experiences places children who are inhibited on a trajectory leading to broad impairment in both unfamiliar settings as well as in situations that are experienced on a daily basis. For example, BI has been posited to predict displays of social withdrawal (solitude) in the preschool setting. In turn, the model suggests, and research has supported the notion that social withdrawal among *familiar* peers predicts deficits in perspective–taking and interpersonal problem-solving skills (e.g., Rubin and Krasnor, 1986; Stewart and Rubin, 1995). These latter deficits, as evidenced in the elementary and middle school years, have been posited, in the conceptual model, and supported in extant research, to predict peer rejection, the consequent development of negative self-appraisals of one’s social competence and relationships, and ultimately, rejection sensitivity and social anxiety during early adolescence (Rubin et al., 1991; Rubin and Chronis-Tuscano, 2021). Indeed, this latter outcome has been supported by research demonstrating that young children who are characterized as being highly inhibited are at increased risk for the later development of social anxiety disorder, which in and of itself is associated with a host of functional impairments throughout adolescence and adulthood (Chronis-Tuscano et al., 2009; Muris et al., 2011; Clauss and Blackford, 2012).

Traditionally, researchers have observed children and their parents in the laboratory setting to identify children high in BI. Furthermore, as noted above, behavioral continuities of BI have been assessed, almost exclusively, within contexts comprising unfamiliar peers (see Rubin et al., 2018 for a relevant review). For example, Kagan (1989) developed a paradigm in which caregivers and their children are placed in an unfamiliar room to engage in unstructured play. While the dyad is playing, an unfamiliar adult enters the room to allow researchers the opportunity to observe children’s reactions, including their hesitancy to interact with the novel adult and toys, frequency of social approach behaviors, and proximity to and interactions with their caregiver (Kagan, 1989; Stifter et al., 1989). In studies of preschool, kindergarten, and elementary school-age children, the consequences of toddler BI have often been assessed by observing children in quartets of unfamiliar peers (e.g., Henderson et al., 2004; Degnan et al., 2008). When laboratory observations are not used, researchers employ a variety of parent- and teacher-report measures to capture BI and conceptually similar constructs in young children (e.g., shyness, social withdrawal, social anxiety), such as the *Behavioral Inhibition Questionnaire* (Bishop et al., 2003), the *Preschool Anxiety Scale* (Spence et al., 2001), and the *Colorado Child Temperament Inventory* (Rowe and Plomin, 1977). However, researchers have noted discrepancies between parent and teacher ratings of BI children, such that both parent and teacher ratings only moderately converge with observational ratings of children’s behavior in the laboratory (Ballespí et al., 2012), thus highlighting the importance of multi-informant and multi-modal measurement. Moreover, given the pivotal role that positive and negative peer interactions play in the aforementioned developmental cascade model toward child social outcomes (Rubin et al., 2009; Rubin and Chronis-Tuscano, 2021), it is imperative to not only observe inhibited children’s reactions in the face of novelty, but also within the peer/social contexts in which their social difficulties may actually ensue.

In one of the few studies to examine associations between laboratory assessments of BI and school-based assessments of social withdrawal, Tarullo et al. (2011) found that compared to preschool-age children high in exuberance (e.g., high activity levels, stimulation-seeking, and risk-seeking), laboratory-identified inhibited preschoolers were observed to experience fewer positive peer interactions, engage in more watching/wandering behavior, interact more with the teacher, and display less positive affect and more anxious/vigilant and sad affect within the familiar setting of the classroom (Tarullo et al., 2011). Although this seminal study highlighted inhibited preschoolers’ unique social experiences within the familiar peer setting, the authors noted that only a relatively small number of children were actually identified as temperamentally inhibited. This disclosure may limit the generalizability and conclusions that can be drawn from the findings. Moreover, the children who were inhibited were compared to a sample of children with highly exuberant temperaments. This latter group may well display markedly different social behaviors/interactions than an unselected sample of children. As such, a replication and extension of these findings is warranted in which a larger sample of inhibited children is compared with an unselected sample of same-age peers within a naturalistic setting.

Also warranted is an examination of the extent to which one of the most frequently used measures of BI, the *Behavioral Inhibition Questionnaire* (BIQ; Bishop et al., 2003), can distinguish between those preschoolers who are identified as highly inhibited from those who represent a “typical” sample vis-a-vis their observed behavior in a school setting populated by familiar peers. Thus, we sought to compare observed and teacher-reported behaviors of children who are behaviorally inhibited with those who are typically developing in their preschool classrooms. We did so by recruiting a reasonably large sample of preschoolers who had been identified, by parent assessments on the BIQ, as highly inhibited and compared their classroom behaviors with an unselected sample of same-age children. Based on prior findings (e.g., Tarullo et al., 2011), we hypothesized that the children identified as highly inhibited would display more solitude as well as less group play compared to their typically developing peers. As previous studies have highlighted low activity levels in children identified as shy and anxious (Poole and Schmidt, 2018), we expected that children identified as inhibited would display less rough-and-tumble play (i.e., playful physical contact, roughhousing, or pretend fighting with peers; Rubin, 1982) compared to their typically developing classmates. Furthermore, we hypothesized that children identified as inhibited would make fewer social initiations to, and receive fewer social initiations from, their classmates and teachers as early inhibition has been shown to predict less social competence and prosociality with peers (Bohlin et al., 2005). We also assessed teacher-reports of child behavior. We hypothesized that preschool teachers would assess the highly inhibited preschoolers as being more solitary, anxious, and excluded by their peers and less prosocial and aggressive relative to their typically developing age-mates.

## 2. Materials and methods

### 2.1. Procedure

Two samples of children were recruited for the study. The first sample comprised a group of children identified as highly behaviorally inhibited (see Sample 1 description below); the second sample comprised a group of children who were matched in age to Sample 1 (see Sample 2 description below). All children fell between the ages of 45–64 months (*n* = 275). They were recruited through community organizations (e.g., schools, daycare centers, pediatrician offices) in the surrounding Washington, DC metropolitan area. Exclusionary criteria included current engagement in anxiety-focused treatment, a diagnosis of autism spectrum disorder or a score at or below the clinical cutoff on the Social Communication Questionnaire (SCQ; Eaves et al., 2006), or a diagnosis (or suspected diagnosis) of selective mutism. Additionally, current enrollment in a preschool/daycare program was required for study participation.

A telephone screen to assess eligibility was completed with parents who expressed interest in participating in the study. A primary parent was identified to complete demographic information and assessments online *via* Qualtrics software. Written informed consent was obtained from all parents. After obtaining informed consent from families, school administrators and teachers were contacted for permission to complete school-based observations of children enrolled in the study. Teachers of participating children also completed questionnaires to assess children’s behavior in the school setting. Trained study personnel conducted 30-min observations of each child during free play in the school setting on each of two separate days. Study materials and procedures were approved by the research team’s university Institutional Review Board. Parents and teachers were compensated for the completion of questionnaires. Data were collected between 2015 to 2020.

### 2.2. Participants

#### 2.2.1. Sample 1 – behaviorally inhibited sample

One hundred thirty children (*n* = 130; *M* = 54 months, *SD* = 5.73) comprised Sample 1. The sample included 68 girls (52.3%) and 62 boys (47.7%). Children from this sample were recruited as part of a larger randomized controlled trial examining early intervention programs for children high in BI (Chronis-Tuscano et al., 2022; ClinicalTrials.gov registration: NCT02308826). The current study uses *baseline* data from this preregistered intervention study. For inclusion in this sample, children had to score in the 85^th^ percentile or above on the *Behavioral Inhibition Questionnaire* (Bishop et al., 2003).

#### 2.2.2. Sample 2 – typically developing sample

One hundred forty-five children (*n* = 145; *M* = 53 months, *SD* = 5.33) were included in Sample 2. The sample comprised 76 girls (52.4%) and 69 boys (47.6%). Children in this sample were recruited for the purpose of comparing the BI sample to typically developing children unselected for BI.

### 2.3. Measures

#### 2.3.1. Demographic variables

Prior to completing the school observations, parents provided demographic information (e.g., sex, age, race, and ethnicity) for themselves and their child (Table 1). Parents also indicated the total time that their child spent in school each week to control for variations in types of school schedules (e.g., full-day vs. extended-day programs).

**Table 1:** Primary parent and child demographic characteristics.

Variable	Sample 1 (*n* = 130)	Sample 2 (*n* = 145)	Combined sample (*N* = 275)
Primary parent	–	–	–
Parent age in years, *M* (*SD*)	38.87 (5.35)	37.79 (5.33)	38.29 (5.37)
Parent relationship to child (% mother)	87.6	89.0	88.0
Parent marital status (%)	–	–	–
Married	89.9	93.1	90.9
Divorced/Separated	4.7	2.8	3.6
Other	5.4	4.2	5.4
Parent race (%)	–	–	–
Asian	17.7	7.6	12.4
Black or African American	13.1	4.8	8.7
White	66.2	78.6	72.7
Other	3.1	8.4	5.8
Hispanic or Latino (%)	6.9	4.9	5.8
Annual household income (USD, %)	–	–	–
$0–$24,999	3.2	0.0	1.8
$25,000–$49,999	3.2	0.0	1.8
$50,000–$74,999	4.0	7.1	5.4
$75,000–$99,999	7.1	6.1	6.7
$100,000–$124,999	17.5	11.2	14.7
$125,000–$149,999	8.7	18.4	13.0
$150,000+	56.3	57.1	56.7
Child	–	–	–
Child age in months, *M* (*SD*)	53.88 (5.73)	52.96 (5.33)	53.37 (5.53)
Child sex (% female)	52.3	52.4	52.4
Child race	–	–	–
Asian	13.1	4.9	8.8
Black or African American	11.5	5.6	8.4
White	50.8	72.2	62.0
Other	24.6	17.4	20.8
Child time in school, *M* (*SD*)*	29.16 (13.44)	26.81 (13.89)	27.97 (13.71)
Time of school observation (% fall)	41.5	37.2	39.3

*Refers to the number of hours child spent in school per week. Sample 1 = Behaviorally inhibited sample; Sample 2 = Typically developing sample.

#### 2.3.2. Behavioral inhibition questionnaire

The *Behavioral Inhibition Questionnaire* (BIQ; Bishop et al., 2003) was used as the primary measure of *parent-reported* child BI. The BIQ is a psychometrically sound (Kim et al., 2011) 30-item caregiver-rating scale that assesses children’s responses to novel situations across six domains: adults, peers, performance demands, novel settings, physical challenges, and parental separation. Parents rated child behavior on a 7-point Likert-type scale from 1 (*Hardly ever*) to 7 (*Almost always*). The BIQ provides a score indicative of social inhibition (comprising the adults, peers, and performance demands subscales) and a score that captures BI in novel and unfamiliar situations (comprising the novel settings, physical challenges, and parental separation subscales). A total BI score can be calculated by summing all items. Scores that fall above 132 (i.e., the top 15% of scores) are within the highly inhibited range based on conceptual models of BI (Kagan et al., 2007). Higher scores reflect more concerns related to BI. Within the current study, reports of internal consistency were high (overall sample α = 0.96, Sample 1 α =0.87, Sample 2 α = 0.95).

#### 2.3.3. Play observation scale

A short form of the *Play Observation Scale* (POS; Rubin, 1982) was used to assess children’s social and non-social behaviors in the classroom setting. Observations began in mid-October so that children had the opportunity to acclimate to the school setting. The POS includes two elements to capture the behaviors of the child being observed (the target child): Time-sampled codes (i.e., mutually exclusive behaviors that are captured within 10-s intervals) and event-sampled codes (i.e., non-mutually exclusive behaviors that were coded each time they occurred). Time-sampled codes included five categories of child’s behavior: reticence (unoccupied and observing/onlooking others from afar); solitary behavior (playing at least three feet away from other children); parallel play (independent play within three feet of other children); group activity (engaging in the same activity as peers or conversing with at least one other child); and teacher interaction (conversing or engaging with a teacher or other adult in the classroom). Also coded were five event-sampled behaviors including anxious behavior (e.g., crying, whining, nail biting, automanipulative activity), positive affect (e.g., laughing), social initiations made *to peers*, social initiations received *from peers*, social initiations made *to teachers*, and social initiations received *from teachers*. To account for minor differences in the time spent observing each child, a proportion was created by dividing the number of 10-s time samples that behavior was coded by the total number of 10-s time samples that each child was observed. Senior research personnel trained staff to reliably collect the in-school POS data. Observers were required to reach high interrater reliability consisting of κ greater than 0.80 with senior research personnel on training videos. Following the completion of training using pre-recorded videos of children’s play in the laboratory, research personnel were required to reach a κ equal to or greater than 0.80 during a live observation session at a local childcare facility affiliated with the university. To control for time of year, observations were dichotomously coded based on the timing during the academic year to either be a “fall” observation (i.e., occurring during the months October through December) or a “spring” observation (i.e., occurring during the months of February through June).

#### 2.3.4. Child behavior scale

The *Child Behavior Scale* (CBS; Ladd and Profilet, 1996) is a teacher questionnaire used to assess social interaction in the school context. Teachers rated children’s behavior on 35 items using a 3-point scale (1: *Does not apply*, 2: *Applies sometimes*, and 3: *Certainly applies*). The CBS comprises six subscales that capture peer aggression, prosocial behavior with peers (e.g., helps other children, empathetic, cooperative with peers, shows concern for moral issues), asocial behavior in the company of peers (e.g., prefers to play alone, keeps peers at a distance, withdraws from peer activities), exclusion by peers (e.g., not much liked by children, ignored by peers, not chosen as a playmate by peers, ridiculed by peers), anxious-fearful behavior, and hyperactive-distractible behavior. Within the current study, reports of internal consistency were acceptable (Cronbach’s α for subscales from the overall sample ranged from α = 0.77–0.90, Sample 1 ranged from α = 0.77–0.92, Sample 2 ranged from α = 0.77–0.87).

#### 2.3.5. Preschool play behavior scale

The *Preschool Play Behavior Scale* (PPBS; Coplan and Rubin, 2001) is an 18-item teacher-report measure designed to capture the reticent behavior (e.g., takes role of onlooker/spectator; wanders around aimlessly; watches/listens to other children without trying to join in; remains alone/unoccupied or staring into space), solitary-passive behavior (e.g., plays alone examining an object/toy; plays alone building things or with other toys; plays alone drawing/painting or doing puzzles; plays alone exploring toys/objects trying to figure out how they work), solitary-active behavior (e.g., engages in pretend play by self; plays make-believe, but not with other children), social play (e.g., talks to other children during play; engages in groups with other children (not just beside them); engages in active conversations with other children), and rough-play (e.g., rough-and-tumble play; engages in playful fighting with other children) of preschool-aged children in the classroom setting. Teachers were instructed to rate child behavior during free play periods from a scale of 1 (*Never*) to 5 (*Very often*). Within the current study, reports of internal consistency were acceptable (Cronbach’s α for subscales from the overall sample ranged from 0.78–0.91, Sample 1 ranged from 0.68–0.95, Sample 2 ranged from 0.64–0.94).

### 2.4. Data analytic plan

Hypotheses were tested using structural equation modeling (SEM) in the *lavaan* package in R (Rosseel, 2012; R Core Team, 2013). A series of regression analyses were run to examine differences in child behavior between the behaviorally inhibited children and the typically developing group for each of the outcome variables. The direct effect of condition on teacher- and parent-reported measures as well as observed behaviors was examined. Robust full-information maximum likelihood was used to handle missing data (Enders, 2001).Theoretically relevant demographic factors [e.g., child sex, child age, total time spent in school, and time of year of the school observations (i.e., fall or spring)] were included as covariates in all analyses.

## 3. Results

### 3.1. Descriptive statistics

Preliminary analyses were conducted using SPSS version 26. Descriptive statistics by sample for all variables of interest are found in Tables 1, 2. Patterns of missingness were examined prior to running analyses. Missingness ranged from 1–19% for teacher-reported variables, and there were no missing data for observed variables.

**Table 2:** Descriptive statistics of variables of interest.

Variable	Sample 1, *M* (*SD*) (*n* = 130)	Sample 2, *M* (*SD*) (*n* = 145)
BIQ Total	153.36 (19.95)	98.13 (28.62)
BIQ Social Inhibition Composite	5.61 (0.80)	3.53 (1.11)
BIQ Adults Subscale	5.65 (1.35)	3.60 (1.48)
BIQ Peers Subscale	5.71 (0.95)	3.63 (1.23)
BIQ Performance Subscale	5.43 (1.14)	3.28 (1.24)
BIQ Novelty Composite	5.12 (0.84)	3.32 (1.09)
BIQ Novel Subscale	4.69 (0.83)	3.08 (0.98)
BIQ Physical Challenges Subscale	3.36 (1.43)	2.51 (1.21)
XBIQ Separation Subscale	5.14 (1.45)	3.18 (1.45)
CBS Peer Aggression Subscale	1.11 (0.27)	1.20 (0.27)
CBS Prosocial Behavior Subscale	2.18 (0.51)	2.35 (0.42)
CBS Asocial Behavior Subscale	1.63 (0.60)	1.42 (0.41)
CBS Exclusion Subscale	1.20 (0.34)	1.19 (0.29)
CBS Anxious-Fearful Subscale	1.43 (0.47)	1.42 (0.47)
CBS Hyperactive-Distractible Subscale	1.25 (0.44)	1.49 (0.56)
POS Time-Sampled Behavior	–	–
Reticence	0.22 (0.16)	0.14 (0.10)
Solitary Behavior	0.23 (0.15)	0.19 (0.13)
Parallel Play	0.22 (0.16)	0.13 (0.10)
Group Activity	0.31 (0.21)	0.42 (0.21)
Teacher Interaction	0.08 (0.08)	0.11 (0.12)
POS Event-Sampled Behavior	–	–
Anxious behavior	0.01 (0.03)	0.01 (0.04)
Positive affect	0.04 (0.04)	0.03 (0.03)
Social initiations *to peers*	0.04 (0.03)	0.04 (0.02)
Social initiations *from peers*	0.01 (0.02)	0.02 (0.02)
Social initiations *to teachers*	0.03 (0.03)	0.03 (0.02)
Social initiations *from teachers*	0.02 (0.02)	0.02 (0.01)
PPBS Reticent Behavior Subscale	2.45 (0.89)	2.19 (0.67)
PPBS Solitary-Passive Behavior Subscale	3.08 (0.78)	2.90 (0.61)
PPBS Solitary-Active Behavior Subscale	2.58 (0.97)	2.55 (0.69)
PPBS Social Play Subscale	3.46 (1.05)	4.07 (0.79)
PPBS Rough Play Subscale	1.90 (1.00)	2.13 (1.04)

BIQ, Behavioral inhibition questionnaire; CBS, Child behavior scale; POS, Play observation scale; PPBS, Preschool play behavior scale. Sample 1 = Behaviorally inhibited sample; Sample 2 = Typically developing sample.

### 3.2. Observational data

#### 3.2.1. Reticence

In the model examining differences in observed child reticence, it was found that the sample of parent-reported BI children exhibited significantly more reticent behavior in their classrooms than children in the typically developing sample (*b* = 0.081, *z* = 4.923, *p* < 0.001). Predictors explained 9.6% of the variance. Child time in school was a significant covariate (*b* = −0.001, *z* = −2.147, *p* < 0.001), suggesting more time in school was related to less reticent behavior for both groups.

#### 3.2.2. Solitary play

In the model examining differences in solitary play between the BI and typical samples, children who were inhibited exhibited significantly more solitary play in their classrooms than typically developing children (*b* = 0.050, *z* = 3.028, *p* = 0.002). Predictors explained 6.5% of the variance. Child age was a significant covariate (*b* = −0.003, *z* = −2.067, *p* = 0.039); older children engaged in less solitary play across both groups.

#### 3.2.3. Parallel play

There were no significant group differences found in observed child parallel play (*b* = 0.019, *z* = 1.455, *p* = 0.146). Predictors explained 5.1% of the variance. Child sex was a significant covariate (*b* = − 0.031, *z* = − 2.452, *p* = 0.014), with boys engaging in significantly less parallel play than girls across groups. Total time spent in school (*b* = − 0.001, *z* = 2.948, *p* = 0.009) was also a significant covariate, with those spending more time in school displaying less parallel play across groups.

#### 3.2.4. Group activity

It was found that children who were behaviorally inhibited exhibited significantly less group play than children who were not inhibited (*b* = −0.123, *z* = −5.214, *p* < 0.001). Predictors explained 13.4% of the variance. Child age (*b* = 0.008, *z* = 3.572, *p* < 0.001) and total time spent in school (*b* = 0.002, *z* = 2.948, *p* = 0.003) were significant covariates, with older children and those spending more time in school engaging in more group play across groups.

#### 3.2.5. Teacher interaction

Children who were highly inhibited exhibited significantly less teacher interaction than typical children (*b* = −0.028, *z* = −2.360, *p* = 0.018). Predictors explained 4.7% of the variance. Child age was a significant covariate (*b* = −0.003, *z* = −1.987, *p* = 0.047); younger children from both groups engaged in more interaction with their teachers.

#### 3.2.6. Event-sampled behaviors

Unexpectedly, in the models examining group differences in event-sampled behaviors, sample was not a significant predictor of observed anxious behavior (*p* = 0.288), positive affect (*p* = 0.642), social initiations made to peers (*p* = 0.349), social initiations received from peers (*p* = 0.126), or social initiations received from teachers (*p* = 0.177).

However, it was found that children who were inhibited exhibited significantly fewer social initiations to teachers than typical children (*b* = −0.029, *z* = −2.151, *p* = 0.031). The predictors explained 4% of the variance in social initiations made to teachers.

### 3.3. Teacher-report comparisons

#### 3.3.1. Preschool play behavior scale

On the PPBS, teachers rated children who were inhibited as engaging in more reticent behavior (*b* = 0.290, *SE* = 0.098, *B* = 0.183, *p* = 0.003) and more solitary activity that involved constructive activity with objects (e.g., puzzle construction; artwork; *b* = 0.183, *SE* = 0.086, *B* = 0.132, *p* = 0.033), than typical children. There were non-significant group differences in teacher ratings of child solitary active play (e.g., running aimlessly around the playroom; *b* = 0.045, *SE* = 0.108, *B* = 0.027, *p* = 0.679). Further, teachers rated typical children as engaging in more rough-and-tumble play (*b* = −0.262, *SE* = 0.116, *B* = −0.127, *p* = 0.024) and more social play involving cooperation between and conversations among peers (*b* = −0.639, *SE* = 0.115, *B* = −0.329, *p* < 0.001), compared to children who were highly inhibited.

#### 3.3.2. Child behavior scale

On the CBS, there were significant differences between groups on several domains. Teachers rated typical children as being more aggressive (*b* = −0.097, *SE* = 0.034, *B* = −0.178, *p* = 0.004) and more prosocial (*b* = −0.172, *SE* = 0.061, *B* = −0.179, *p* = 0.005) than children who were inhibited. Additionally, teachers rated children who were inhibited as being more asocial than typical children (*b* = 0.185, *SE* = 0.063, *B* = 0.183, *p* = 0.003). However, there were no significant group differences in teacher ratings of child anxiety (*b* = 0.010, *SE* = 0.062, *B* = 0.011, *p* = 0.870) or teacher ratings of child exclusion (*b* = 0.019, *SE* = 0.040, *B* = 0.030, *p* = 0.640).

## 4. Discussion

Although researchers have long been reporting behavioral differences between extremely inhibited and typical children when these groups are observed in an unfamiliar setting (e.g., the laboratory) and in the company of unfamiliar peers (see Rubin et al., 2018 for a review), few studies have extended this research to the naturalistic and familiar context of the school. Moving beyond observations in laboratory settings to understand how inhibited children function in familiar contexts is essential if one is to establish support for the conceptual conjecture that BI in early childhood is linked to the display of solitude in familiar settings that, in turn, predicts subsequent difficulties in the peer group (rejection; victimization), negative thoughts and feelings about the self, and ultimately to anxiety (and more specifically, social anxiety; Rubin et al., 2009). Although the ability to regulate emotions and behavior in novel situations and contexts is crucial to optimal child development, continued adaptability in everyday social contexts is highly significant to overall child functioning. Thus, the primary aim of the current study was to examine links between a reliable and valid index of parent-reported BI (Bishop et al., 2003; Broeren and Muris, 2010; Kim et al., 2011) and observed and teacher-reported child behaviors in the preschool setting with familiar peers.

In accordance with our hypotheses and previous research, our findings revealed that children identified as highly inhibited on the parent-report BIQ were observed to engage in more reticent behavior and solitary play and in less social interaction with peers and teachers than a comparison group of typical (non-BI) age-mates. Indeed, both observational data and teacher-reports indicated that inhibited children evidenced significantly more reticence and solitude (i.e., unoccupied, observing/onlooking others from afar; solitary play) in the school setting compared to their same-aged typical peers. These findings are in accord with those reported by Tarullo et al. (2011) in a study of a much smaller sample of extremely inhibited children which was compared with a group of highly exuberant preschoolers. When conceptualized within the broader literature pertaining to laboratory-based observations of inhibited children’s behaviors, our findings indicated not only that inhibited preschoolers demonstrated significantly more unoccupied/onlooker behaviors (i.e., reticence; Coplan et al., 1994), but also more solitary activity compared to their typically developing peers.

It is notable that children in the inhibited sample did not engage in less parallel play (i.e., independent play within three feet of other children) compared to their typically developing counterparts. Researchers have suggested that parallel play may be a necessary step that allows inhibited children to progress from watching others from afar or choosing to express solitude to eventually approaching others in an effort to engage in social interaction (Bakeman and Brownlee, 1980; Asendorpf, 1991). Perhaps a different observational taxonomy and the use of such statistical methods as sequential analyses may allow researchers to examine whether those inhibited children who gradually come to engage others in social interaction do, indeed, display a sequential process of observing others from afar, to approaching others and quiescently marking territory in close proximity to specific peers, to requesting that they join the activities of the desired peers.

In general, the teacher reports supported that which was observed. Thus, teachers indicated that children identified as behaviorally inhibited evidenced significantly more reticence and solitary passive (e.g., quiescent object exploration and construction) activity. No group differences emerged with regard to teacher-reported solitary active play. Given that the latter form of solitude is rather infrequently displayed during preschool free play (e.g., Rubin, 1982) the non-significant between-group difference is unsurprising.

Contrary to our hypotheses, children in the behaviorally inhibited sample did not make or receive fewer bids for social interactions than their typically developing peers. Despite these non-significant differences, it may have been that the preschoolers identified as inhibited were approaching their age-mates in a less than competent manner, thereby negating the possibility of engaging with peers in cooperative, group-oriented play. Unfortunately, our observational coding taxonomy did not distinguish between positive and negative social overtures to (and from) peers. However, in previous studies, researchers have established that inhibited and socially withdrawn preschoolers are less socially competent than their typically developing same-age peers (e.g., Rubin et al., 1991; Bohlin et al., 2005). For example, inhibited and withdrawn children have been observed to be less successful than their typically developing age-mates in being able to meet their social goals (Rubin and Krasnor, 1986; Stewart and Rubin, 1995). Furthermore, inhibited and withdrawn preschoolers have been found to be less able than their more sociable age-mates to generate competent and flexible strategies to join others in play or to establish friendships (Rubin and Krasnor, 1986). Perhaps these latter difficulties may explain why the BI children in the present sample were unable to successfully initiate sustained social interaction or to capitalize on opportunities offered by peers to engage in social play.

Lastly, it may have been possible that the bids for social interaction received by inhibited children were not for the purpose of initiating coordinated and positive social play. Thus, despite the lack of between group differences in teacher ratings of peer exclusion, it is possible that more subtle negative peer interactions are not as noticeable to teachers at this developmental stage. Notably, preschool teachers are more likely to notice physical aggression and defiance as forms of bullying, but often overlook bullying that occurs in verbal and relational forms (Tepetaş et al., 2010). Furthermore, as the children in the current study were in their first years of school, they may not have reached the point at which solitary behavior is considered, by peers, to be abnormal (Younger et al., 1993). Thus, the BI preschoolers who expressed reticent and solitary behavior in the classroom may not have been viewed as being “easy targets” for peer victimization, exclusion, and rejection (Ladd, 2006; Rubin et al., 2009) as is the case for older, elementary school-aged socially withdrawn children. To further pinpoint the emergence of this developmental transactional process, researchers would do well to examine the content and quality of inhibited and socially withdrawn children’s peer interactions across time.

As expected, inhibited children were reported, by teachers, to engage in significantly less rough-and-tumble play compared to their typically developing peers. Significantly, rough-and-tumble play can be distinguished from acts of aggression in that the former is not considered to involve a goal to harm the play partner (Pellegrini, 2002). Indeed, there is a growing body of research regarding the benefits of “adventurous play” for children. More specifically, play in which children have the opportunity to take developmentally appropriate risks in a playful manner has been linked with reduced social anxiety later on in childhood (Majdandžić et al., 2018). Thus, there are clear potential benefits for supporting inhibited children’s adventurous play in an effort to mitigate their already elevated risk for later social anxiety. Teachers play a critical role in increasing children’s access to adventurous play, and several school-based interventions have been developed with the goal of facilitating opportunities for risk and challenge in children’s play (see Nesbit et al., 2021 for a systematic review). Nevertheless, further research is needed to identify the most effective ways of supporting children’s adventurous play and eliminating school-related barriers to implementing related interventions.

With regard to teacher interactions, children in the behaviorally inhibited sample spent significantly less time in play or in conversation with their teachers compared to their typically developing peers. While teachers initiated social interactions with children in both samples at similar frequencies, inhibited children made significantly fewer initiations to their teachers. Over time, if inhibited children lack the repertoire of social skills to support effective communication with their teachers, they may face challenges advocating for their needs to be met in the classroom. Difficulties vocalizing their needs to teachers may also make inhibited children more susceptible to peer victimization across the school years (Rubin et al., 2009). Indeed, researchers have shown that inhibited children lack closeness in their relationships with their teachers, even when they are engaged in fewer personal conflicts in the classroom setting (Rudasill et al., 2006; Thijs and Koomen, 2009). Given the significant role of positive teacher-child relationships in supporting both social and academic success (Rudasill et al., 2006), it is of prime importance to improve behaviorally inhibited children’s ability to connect with, and benefit from, their relationships with their teachers.

Our findings have several implications for prevention/intervention efforts for inhibited young children. First, the significant differences between inhibited and typically developing children highlight tangible opportunities for *early* intervention efforts. When inhibited children engage in less social interaction within their first years of school, they naturally encounter fewer opportunities to gain knowledge of social relationships and utilize social skills (Rubin et al., 2009). To disrupt this negative developmental process from unfolding, intervention programs would benefit from targeting inhibited children’s social skills, with the goal to generalize the learned skills to the school setting, and ultimately increase positive peer and teacher interactions. Along with age-appropriate play and social skills, it may be particularly beneficial for such programs to equip inhibited children with assertive communication skills to ensure that their needs are not overlooked. Importantly, engagement in reticent or solitary-passive play may not, in and of itself, warrant intervention to mitigate the risk for developing anxiety; other factors that may underlie the expression of these behaviors must be taken into account (Coplan and Rubin, 2001). Such other child factors include the ability to regulate emotion and the ability to understand the perspectives and feelings of others. These factors must be assessed to determine whether, or which type of intervention is necessary to best support children’s social and emotional development. Furthermore, teachers can play an important role in scaffolding inhibited children’s social development in the classroom. In the current sample, inhibited children engaged in less group activity with peers and less teacher interaction compared to their typically developing. Teachers and other educational staff may benefit from evidence-based strategies to engage inhibited children in both adult and peer interactions. To facilitate inhibited children’s social skill development and offer naturalistic opportunities for sustained social interaction, teachers may benefit from intervention/prevention efforts that incorporate social skills and associated group-based activities into the regular classroom curriculum (Coplan and Rudasill, 2016).

The current study expands on previous work in several ways. First, we discovered, for the first time, that the oft-used BIQ allows a distinction to be made between the classroom free-play behaviors of young, inhibited children and their uninhibited counterparts. Knowing that the parent-reported BIQ can distinguish between inhibited and uninhibited children’s behaviors in both unfamiliar and familiar settings will be useful in the screening of children in need of intervention (e.g., *The Turtle Program* – Chronis-Tuscano et al., 2022; *The Cool Little Kids Program* – Rapee et al., 2005).

Relatedly, by comparing children with elevated BI with a sample of typically developing children in the school setting, we were able to gain insight into inhibited children’s behaviors in the context of their familiar peers, rather than in an unfamiliar laboratory setting comprising groups of unacquainted children. Although BI is characterized by wariness in the context of novelty, examining children in their natural settings provides opportunities to better understand the ways in which inhibited youth’s socioemotional development can be optimally supported across settings.

Furthermore, multiple informants’ reports (teacher and parent) were utilized in the current study to characterize the children’s behaviors, along with objective school-based observations. A multi-informant approach is essential, as child behaviors have repeatedly been shown to vary across environments and caregivers (De Los Reyes et al., 2013).

It is important to acknowledge the limitations of the current study. First, many of the children from both samples attended highly resourced preschools. As these settings are not representative of all preschools, it will be important for future researchers to take the classroom context and curriculum into account to ensure that various school formats are incorporated into the sample. Second, studies would benefit from including a measure of classroom emotional climate, which has been shown to buffer against socially withdrawn youths’ experiences of peer rejection and victimization (Gazelle, 2006). Third, a measure of children’s language development was not included as part of the current study. While it is possible that language delays may account for fewer social initiations to peers and teachers (e.g., Coplan and Weeks, 2009), word approximations, single words, phrases, and sentences are all sufficient for a code of social initiation to others as part of the Play Observation Scale (Rubin, 1982). Moreover, children are able to engage in collaborative group play (i.e., with a common goal or purpose) without verbal communication to receive a code of “group play” on the Play Observation Scale. Nevertheless, in the future, researchers should incorporate measures of language skills when evaluating behavioral inhibition to disentangle the constructs of verbal communication and sociality. Finally, children in the current study were measured at one timepoint in their school classrooms. As peer experiences in the classroom setting may impact behavior across time (Almas et al., 2011), in the future, would do well to assess child behavior at multiple timepoints.

In sum, the goal of the current study was to compare the in-school behaviors of two distinct groups of preschoolers – one comprising typical children and the other comprising children identified as dispositionally behaviorally inhibited. The study was designed to establish whether BI was associated with the display of solitude in the company of familiar peers. Indices of BI were drawn from parents and trained observers. Findings from the present study suggested that children high in BI differed from typically developing children in the extent to which they were observed to engage in social reticence and solitude in the school setting. While inhibited children engaged in more reticent and solitary behaviors and less group-based interactive play, they received similar amounts of social initiations from their classmates. The findings provide evidence for the social challenges inhibited children face in *familiar* peer contexts, and indicate that young, inhibited children may have difficulties capitalizing on their peers’ advances to foster social connection. These findings have several implications for early intervention and prevention efforts, as children high in BI may require additional support from parents and teachers to develop social skills through peer interaction.

## Data availability statement

The datasets presented in this article are not readily available because of ethical and privacy restrictions. Requests to access the datasets should be directed to the corresponding author.

## Ethics statement

The studies involving human participants were reviewed and approved by Institutional Review Board, University of Maryland-College Park. Written informed consent to participate in this study was provided by the participants’ legal guardian/next of kin.

## Author contributions

LD, DN, KS, and KR contributed to the conceptualization and design of the study. LD, DN, and KS organized the database. DN and KS performed the statistical analyses. LD, DN, and KR wrote the first draft of the manuscript. NW, SP, HF, CD, and AC-T reviewed and edited this manuscript. LD, DN, KS, SP, HF, and CD were responsible for project administration. KR and AC-T designed the original study and acquired the funding for this project. All authors contributed to the article and approved the submitted version.

## Funding

This project was funded by a grant from NIH R01MH103253-01 awarded to AC-T and KR.

## Conflict of interest

The authors declare that the research was conducted in the absence of any commercial or financial relationships that could be construed as a potential conflict of interest.

## Publisher’s note

All claims expressed in this article are solely those of the authors and do not necessarily represent those of their affiliated organizations, or those of the publisher, the editors and the reviewers. Any product that may be evaluated in this article, or claim that may be made by its manufacturer, is not guaranteed or endorsed by the publisher.
